# The Implications of Atlantic Salmon (*Salmo salar* L.) Fatty Acid Profiles for Their Thiamine Status

**DOI:** 10.1002/ece3.70478

**Published:** 2024-10-25

**Authors:** Vittoria Todisco, Marc M. Hauber, Michael T. Brett, Charlotte Axén, Kjetil Hindar, Petter Tibblin, Samuel Hylander

**Affiliations:** ^1^ Centre for Ecology and Evolution in Microbial Model Systems (EEMiS) Linnaeus University Kalmar Sweden; ^2^ Department of Civil and Environmental Engineering (CEE) University of Washington Seattle Washington USA; ^3^ Swedish Veterinary Agency (SVA) Uppsala Sweden; ^4^ Norwegian Institute for Nature Research (NINA) Trondheim Norway

**Keywords:** Atlantic salmon, DHA, diet history, fatty acids, M74, thiamin, thiamine

## Abstract

Thiamine deficiency is an ongoing issue across the Northern Hemisphere, causing reproductive failure in multiple salmonid populations. In the Baltic Sea, a large brackish water system in northern Europe, previous research has suggested that this deficiency is associated with lipid‐rich diets with a high proportion of docosahexaenoic acid (DHA, 22:6n‐3). The mechanism proposed is that a diet abundant in highly unsaturated fatty acids, such as DHA, depletes thiamine as an antioxidant defense in adult salmonids, rather than allocating thiamine to the offspring. In light of this existing hypothesis, we here explore the relationship between diet history and the related fatty acid (FA), profiles, and thiamine status of Atlantic salmon (*Salmo salar* L.) in three systems: the Baltic Sea, the North Atlantic Ocean, and Lake Vänern. Atlantic salmon inhabiting each system is known to have unique feeding histories and thiamine status. Our results showed that despite extensive sampling effort and distinct FA profiles, indicative of their diverse diets, there were no correlations between any FAs, including DHA, and the thiamine status of these populations. This finding does not support the above‐mentioned hypothesis that diets rich in easily oxidized FAs would lead to lower thiamine concentrations in salmon tissues. Additionally, we found that changes in the salmon FA profiles throughout their life cycle are consistent for both low‐thiamine populations from the Baltic Sea and medium‐thiamine populations from North Atlantic Ocean, suggesting that these changes might not be involved in thiamine deficiency development.

## Graphical abstract

Thiamine deficiency, linked to reproductive failure in salmonids, is a significant issue in the Northern Hemisphere, with previous research suggesting it is caused by lipid‐rich diets high in docosahexaenoic acid (DHA). However, our study across three distinct systems—the Baltic Sea, the North Atlantic Ocean, and Lake Vänern—found no correlation between fatty acid (FA) profiles, including DHA, and thiamine status, contradicting the hypothesis that diets rich in easily oxidized FAs lead to lower thiamine concentrations. Furthermore, changes in salmon FA profiles throughout their life cycle were consistent across both low‐thiamine and medium‐thiamine populations, suggesting these changes do not contribute to thiamine deficiency development.

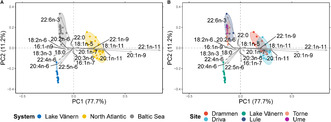

## 1 Introduction

Fatty acids (FAs), the main components of lipids, play a pivotal role in the biological processes of all living organisms. In addition to their structural function in cell membranes, they also serve as one of the major sources of energy for animals (Sargent, Tocher, and Bell 2003). This energy is produced in the form of ATP through a well‐established metabolic pathway occurring in mitochondria known as β‐oxidation that has also been described in fish (Tocher 2003). The energy density of dietary lipids is the highest among all macronutrients (i.e., lipids, proteins and carbohydrates), and therefore represents the most efficient macromolecule for maximizing energy intake for fish (Bureau, Kaushik, and Cho 2003; Glencross 2009). Consequently, FAs constitute the preferred and most important source of metabolic energy in fish for growth in all life stages and during reproduction (Sargent, Tocher, and Bell 2003; Tocher 2010). Essential FAs (EFAs), which are crucial for promoting fish growth and overall health (Sargent, Tocher, and Bell 2003), cannot be synthesized *de‐novo* by animals and must be obtained through their diet (Parrish 2009). In aquatic ecosystems, the requirements for EFAs are species and life stage specific (Watanabe 1982; Ruyter et al. 2000; Glencross et al. 2002). The most important EFAs include the long‐chain omega‐6 and omega‐3 FAs and especially 20:4n‐6 (arachidonic acid, ARA), 20:5n‐3 (eicosapentaenoic acid, EPA), and 22:6n‐3 (docosahexaenoic acid, DHA). Given the physiological significance attributed to EPA and DHA, fish exhibit strong selective retention of these EFAs relative to their diets (Arts and Kohler 2009; Parrish 2009; Bandara et al. 2023). To some extent, fish can synthesize these long‐chain polyunsaturated fatty acids (LCPUFAs) by elongation of shorter FA precursors (such as 18:3n‐3, α‐linolenic acid, ALA; Turchini et al. (2022)), especially in freshwater environments (Sargent, Tocher, and Bell 2003; Leaver et al. 2008). Nevertheless they still rely heavily on direct dietary supply of these FAs for their optimal growth and physiological state. EFAs are synthesized *de‐novo* by primary producers, especially diatoms and cryptophytes, and reach higher trophic levels through grazing and predation (Brett and Müller‐Navarra 1997). Based on the principle that most dietary FAs are conserved and transferred from primary producers to higher trophic levels, FAs have been used as trophic markers (Dalsgaard et al. 2003; Graeve and Greenacre 2020) thereby providing, to some degree, insights into trophic interactions and dietary history. Hence, the FA profiles in fish tissues reflect their diet (Papadimitraki, Maar, and Jónasdóttir 2023); for example, long‐chain monounsaturated fatty acids (MUFAs) such as 20:1n‐9 and 22:1n‐11 are indicators of lipid‐rich copepod or mysid prey items (Dalsgaard et al. 2003; Stowasser, Pond, and Collins 2009), whereas 18:1n‐9 (oleic acid) is instead a general indicator of carnivory (Sargent and Falk‐Petersen 1981; Falk‐Petersen et al. 2000).

Another essential component in the nutritional profile of an organism is represented by the vitamins. Here, we focus on thiamine, also known as thiamin and vitamin B_1_ (Lonsdale 2006). Many salmonid populations in the Northern Hemisphere have been affected by thiamine deficiency, a pathology that causes health impairment and, in severe cases, mortality of adults (Amcoff et al. 1998), as well as reduced offspring survival (Bylund and Lerche 1995; Fitzsimons 1995; Ketola et al. 2000; Brown et al. 2005). For example, during the 1990s, the incidence of M74 syndrome (Börjeson et al. 1999), which results in high mortality among yolk‐sac fry from thiamine‐deficient female salmon, reached 60%–100% in most Swedish rivers (Majaneva et al. 2020). This deficiency in salmonids has been associated with the consumption of prey fish with high thiaminase—a thiamine‐degrading enzyme—activity in the Great Lakes region (Fisher et al. 1996; Honeyfield et al. 2005). Conversely, in the Baltic Sea, one of the main hypothesized causes of thiamine deficiency in Atlantic salmon (*Salmo salar* L.), hereafter called salmon, is a shift in diet toward lipid‐rich prey fish with a high proportion of omega‐3 PUFAs (Keinänen et al. 2012, 2017, 2018, 2022; Vuorinen et al. 2020). The hypothesized mechanism leading to thiamine deficiency is that the requirement for thiamine increases when salmon feed on high‐energy diets during the adult feeding phase before returning to their natal river for reproduction. Therefore, thiamine reduction is proposed to be related to the peroxidation of susceptible FAs, with omega‐3 PUFAs among the most susceptible (Kjær et al. 2008) and the subsequent usage of thiamine itself as an antioxidant defense. The antioxidant properties of thiamine have been demonstrated by Lukienko et al. (2000): in their in vitro experiment, thiamine inhibited lipid peroxidation and free radical oxidation of oleic acid in rat liver cells. However, the potential of thiamine as antioxidant in fish cells and against oxidative stress related to omega‐3 PUFA peroxidation has not yet been demonstrated. Moreover, the mechanism by which thiamine would be used as a preferential antioxidant compared to more established antioxidant systems remains unresolved.

In this study, we use salmon as a model organism to understand how diet history and, therefore, the related FA profiles influence their thiamine status. Our approach includes first a comparison of the FA profiles in salmon across three distinct systems: the Baltic Sea, the North Atlantic Ocean, and Lake Vänern. These three systems are known to have salmon populations with unique diets and different thiamine status (Todisco et al. 2024). Given the divergent prey items of salmon among these systems, we hypothesize that their distinctive feeding histories results in system‐specific FA profiles in their muscle tissues. Additionally, we investigate the potential effect of migration and starvation on the FA profiles in salmon. These physiological challenges when salmon approach reproduction are examples of other factors that might affect FA profiles along with the diet history. Finally, we test if low thiamine status is related to specific FA profiles and in particular to high concentrations of omega‐3 PUFAs according to the current lipid hypothesis (Keinänen et al. 2012, 2017, 2018, 2022; Vuorinen et al. 2020).

## 2 Materials and Methods

### 2.1 Salmon Sampling and Sample Collection

The 102 samples included in the present study are a subset of the samples originally collected by Todisco et al. (2024). Briefly, salmon were captured in 2021 during three adult life stages, i.e. (1) during the marine feeding phase (only in the Baltic Sea) in spring, (2) upon arrival to their natal river after sea migration in summer (river mouth sites), and (3) at reproduction sites upstream in the river during fall. Salmon were caught in the Southern Baltic Sea representing their main feeding area as well as at the river mouths of Torne, Ume, and Lule rivers. Samples were also collected from rivers draining into the North Atlantic Ocean, specifically in Driva and Drammen river mouths in Norway. One landlocked salmon population was sampled in Lake Vänern before their migration upstream into the affluent river Klarälven. In Lule, Drammen, and Driva rivers salmon were also sampled upstream during the spawning phase in fall. In these rivers, sampling during reproduction was possible because the sampling sites were located at salmon hatcheries, where migrating salmon are caught and kept for restocking purposes. Only females are included in this study and a summary of sampling locations, number of individuals, and life stage is presented in Table 1.

**TABLE 1:** Summary of the sampling occasions.

System	Area	Site	Number of individuals	Sampling date (mm/dd/yyyy)	Life stage
Baltic Sea	Southern Baltic Sea		12	March–May 2021	Actively Feeding
	Torne river	River mouth	11	6/17/2021	After Sea Migration
	Lule river	River mouth	11	7/5/2021	After Sea Migration
		Upstream	15	10/18/2021	Actively Spawning
	Ume river	River mouth	3	7/8/2021	After Sea Migration
North Atlantic Ocean	Drammen	River mouth (Oslo Fjord area)	11	6/21/2021	After Sea Migration
		Upstream	8	11/4/2021	Actively Spawning
	Driva	River mouth (Tingvoll Fjord area)	13	7/13/2021	After Sea Migration
		Upstream	4	9/27/2021	Actively Spawning
Lake Vänern	Lake Vänern	In lake at river mouth	14	7/13/2021	After Lake Migration

*Note:* The sampled salmon populations are divided into the above‐mentioned systems: Baltic Sea, North Atlantic Ocean, and Lake Vänern, and the river, lake, or open sea locations where they were caught are indicated in the “Area” column. The “Site” column indicates the specific part of the river where the fish were caught and sampled. The date of sampling is displayed as the month, day, and year (mm/dd/yy) and the life stage is reported as well.

During sampling fish were stunned with a blow to the head and immediately weighed (kg), followed by exsanguination by drawing 10–25 mL of blood combined with a cut through at least one gill arch within 30–60 s from stunning. Then the fish were photographed to document the external status after which total length (cm) was measured. Fulton's condition factor (CF) was calculated as 10^5^ × weight (kg) × (total length (cm))^−3^. Mean total length, weight, and CF are summarized in Appendix S1.

At least 20 g of proximal dorsal muscle was dissected, vacuum packed and immediately placed on dry ice or in a portable freezer (−80°C, Stirling Ultracold ULT25NEU). The samples were transported to the lab and then stored at −80°C until analysis.

### 2.2 Ethics Statement

Sampling was performed abiding to all applicable international, national, and/or institutional guidelines for the care and use of animals. Ethical permission for sampling in the Baltic Sea and in Swedish rivers was granted by the Ethical Committee of Animal Experiments of the Swedish Board of Agriculture (Jordbruksverket) in Linköping (Dnr 16867‐2018) and by the Ethical Committee of Animal Experiments of the Swedish Board of Agriculture (Jordbruksverket) in Uppsala (Dnr 5.8.18‐06256/2019). Wild salmon were sampled with the permission from the Swedish Agency for Marine and Water Management (HaV, (Dnr 2713‐2020)). Furthermore, sampling the Swedish rivers were granted by HaV (Torne river, Dnr 1876‐2021 and 1892‐2021) and the county boards of Västerbotten (Ume river, Dnr 621‐6241‐2021), and Värmland (Klarälven, Dnr 621‐5715‐2021). Sampling in Lule river mouth was performed during the designated fishing window not requiring any permit and Vattenfall authorizied fishing in the upstream area. Permissions to catch, sample, and exsanguinate fish from Drammen, and Driva rivers were granted by the county officers of Oslo and Viken, Møre and Romsdal, and Vestfold and Telemark (Refs. 2021/8912, 2021/3310, 2021/4432).

### 2.3 Fatty Acid Extraction and Quantification

From the dorsal muscle dissected during sampling, approximately 1 g was freeze‐dried and stored at −80°C until analysis. The FAs extraction was performed following Folch, Lees, and Sloane Stanley (1957), with minor modifications. This method allows the transformation of FAs into fatty acid methyl esters (FAMEs) by acid‐catalyzed transesterification. Three sets of 15 mL glass tubes were burn‐cleaned and dried and labeled set‐A, set‐B, and set‐C. About 3 mg of each sample was weighed into the set‐A tubes using one technical replicate per fish. A volume of 4 mL of chloroform‐methanol solution (2:1 volume ratio) was added to each tube with 1 mL of MilliQ water. The tubes were then flushed under a nitrogen (N_2_) gas stream before closing the samples to avoid the presence of oxygen in the tubes, followed by sonication in ice for 10 min, vortexing for 10 s, and centrifuging for 5 min at 1500 rpm (Thermo Sorvall Legend RT, max. rotor radius 19.2 cm). The lower phase was transferred into the set‐B tubes. The extraction was repeated with 2 mL of chloroform in the set‐A tubes and the lower phase was combined with the previously collected one in the set‐B tubes. The chloroform was evaporated under N_2_ flux to near dryness, and 1.3 mL of toluene was added to the set‐B tubes. Two mL of 1% sulfuric acid (H_2_SO_4_) in methanol was added to the tubes and uniform mixing was ensured by vortexing for about 10 s. The tubes were then placed in a water bath for 90 min at 90°C. The tubes were let to cool down for a few minutes before 1.5 mL of MilliQ water and 4 mL of hexane were added to ensure extraction of the FAMEs. The samples were vortexed for 10 s and centrifuged for 2 min at 1500 rpm (Thermo Sorvall Legend RT, max. rotor radius 19.2 cm). The upper phase was carefully transferred into the set‐C tubes and the FAMEs were extracted a second time by adding 4 mL of hexane to the set‐B tubes, vortexing for 10 s, and centrifuging for 2 min at 1500 rpm (Thermo Sorvall Legend RT, max. rotor radius 19.2 cm). The upper phase was transferred in the set‐C tubes. The extracted FAMEs were evaporated to near dryness and 600 μL of hexane were added as a solvent. The mix was transferred to 2 mL sample glass vials and the last step was repeated with an additional 500 μL of hexane. The obtained FAMEs were run through a gas chromatograph coupled with a flame ionization detector (GC‐FID; Hewlett Packard HP6890) with an Agilent DB‐23 column (30 m × 0.25 mm × 0.15 μm). Helium was used as a carrier gas with an average velocity of 25 cm s^−1^. A subset of five representative samples (one for each system and life stage) was run with a gas chromatogram–mass spectrometer (GC‐MS; Shimadzu QP2010 Plus) using the same column and temperature program to confirm peak identifications. Individual FAMEs were identified based on their mass spectra and quantified using a dilution series of a FA standard mixture (GLC‐68D, Nu Chek Prep.) with the following formula:

$$\text{Concentration}\left(\frac{\mathrm{mg}\;\mathrm{FA}}{\mathrm{mg}\;\text{Sample}}\right)=\left(\frac{\text{Peak}\;\text{Area}\times \text{Constant}\times \text{Dilution}\;\text{factor}}{\mathrm{mg}\;\text{of}\;\text{Sample}}\right)\times \text{Hexane}\;\text{Volume}\;\left(\mathrm{mL}\right)$$

The constant, 0.0000656, converts the peak surface area, the so‐called peak area in the formula, in the chromatograph (which is instrument, method, and column specific) to a mass of a given FA. To convert that FA mass to mg mg^−1^ units this equation also considers dilution (i.e., the volume of hexane used) and the dry mass of the tissue sample. The dilution factor was 1 except if the chromatograms showed overlapping peaks due to high FA concentrations. In this latter case, the sample was diluted 3:1 using hexane and analyzed again with the Gas Chromatograph. The concentrations were then converted to mg g^−1^ by multiplying the concentrations in mg mg^−1^ by 1000 mg g^−1^. When proportions were used, they were calculated as the ratio between the concentration of a given FA relative to the total FAs.

### 2.4 Thiamine Analysis

Thiamine was analyzed in the same dorsal muscle samples according to Brown, Honeyfield, and Vandenbyllaardt (1998) with modifications according to Vuorinen et al. (2002), Futia et al. (2017) and Futia et al. (2019) and analytical procedures were described in detail by Todisco et al. (2024). In short, 0.5 g of muscle tissue was homogenized with 2% trichloroacetic acid and incubated at 100°C. After centrifugation at 14,000 *g*, the supernatants were washed with a solution of ethyl acetate: hexane (3:2 volume ratio). A volume of 850 μL of the lower phase was mixed with 150 μL of dye (0.1% K_3_Fe(CN)_6_ in 1.2 M NaOH solution) and filtered through 0.45 μm PTFE/PP filters into sample glass vials. Standard solutions for the three vitamers of thiamine (free thiamine [TF], thiamine monophosphate [TMP], and thiamine diphosphate [TDP]) were prepared and aliquoted in a five‐points standard series. TF, TMP, and TDP were quantified with the Hitachi Chromaster HPLC system using a Hamilton PRP‐1 column (5 μm particle size, 4.1 mm [I.D.] × 150 mm) and a fluorescence detector (excitation wavelength 375 nm, emission wavelength 433 nm). The amounts of the three vitamers were then summed and normalized by wet weight to total thiamine (Ttot) using the unit nmol g^−1^ wet weight. Here we use a subset of thiamine concentrations published in Todisco et al. (2024) to relate to the salmon FA profiles.

### 2.5 Statistical Analyses

Statistical analyses and graphics were performed using R, version 4.2.1 (R Core Team 2021) and its extension packages *vegan* for principal component analysis (PCA) (Oksanen et al. 2023), *multcomp* for post hoc multicomparison (Hothorn, Bretz, and Westfall 2008), and *ggplot2* for graphics (Wickham, Chang, and Wickham 2016). Variations of the FA profiles among systems and among life stages within each system were investigated using PCA. In these cases, FAs data expressed as proportions were used and they were transformed using the Hellinger transformation prior to the analyses to handle the presence of zeros in the data set. Subsequently, permutational multivariate analysis of variance (PERMANOVA) was performed to assess differences in FA profiles between the systems and between life stages within systems using the function *adonis2* from *vegan* package. The models used system or life stage as the predictor variable and the FAs data as response variable. PCA was also used to transform the full FA dataset into a smaller set of new variables (components). The scores of these components were then used to study if the FA profiles in components 1–3 were related to the thiamine concentrations. Principal component loadings were used to interpret the scores of the components. FAs data expressed as concentration in mg g^−1^ were used in the post hoc multicomparisons and correlations. The correlation coefficients (rho) were calculated using Spearman's rank correlation. If structural categories were used, the FAs were divided into: saturated FAs (SFAs), when no double bonds were present in the carbon chain; MUFAs, when one double bond was present in the carbon chain; PUFAs, when multiple double bonds were present in the carbon chain; and LCPUFAs, when the carbon chain contained multiple double bonds and was 20 or more carbon atoms long.

## 3 Results

### 3.1 Differences in FA Profile among Systems and During Different Life Stages within Systems

**FIGURE 1:**
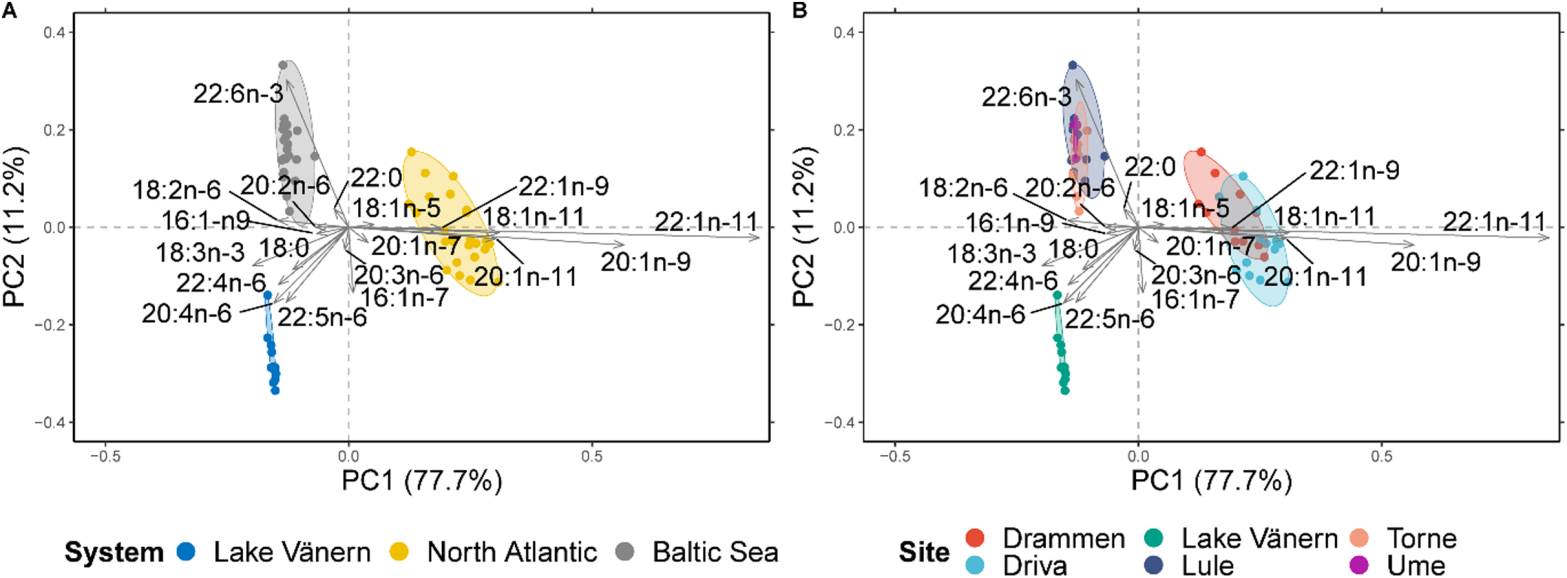
Principal component analysis (PCA) with principal components 1 and 2 and their respective percentage of the explained variance. The reported FAs explain at least 70% of the variation in the samples and they are indicated by the light gray arrows. Only samples of the after sea or lake migration life stage are included (*n* = 63). Different colors indicate (A) different systems; (B) each river.

**FIGURE 2:**
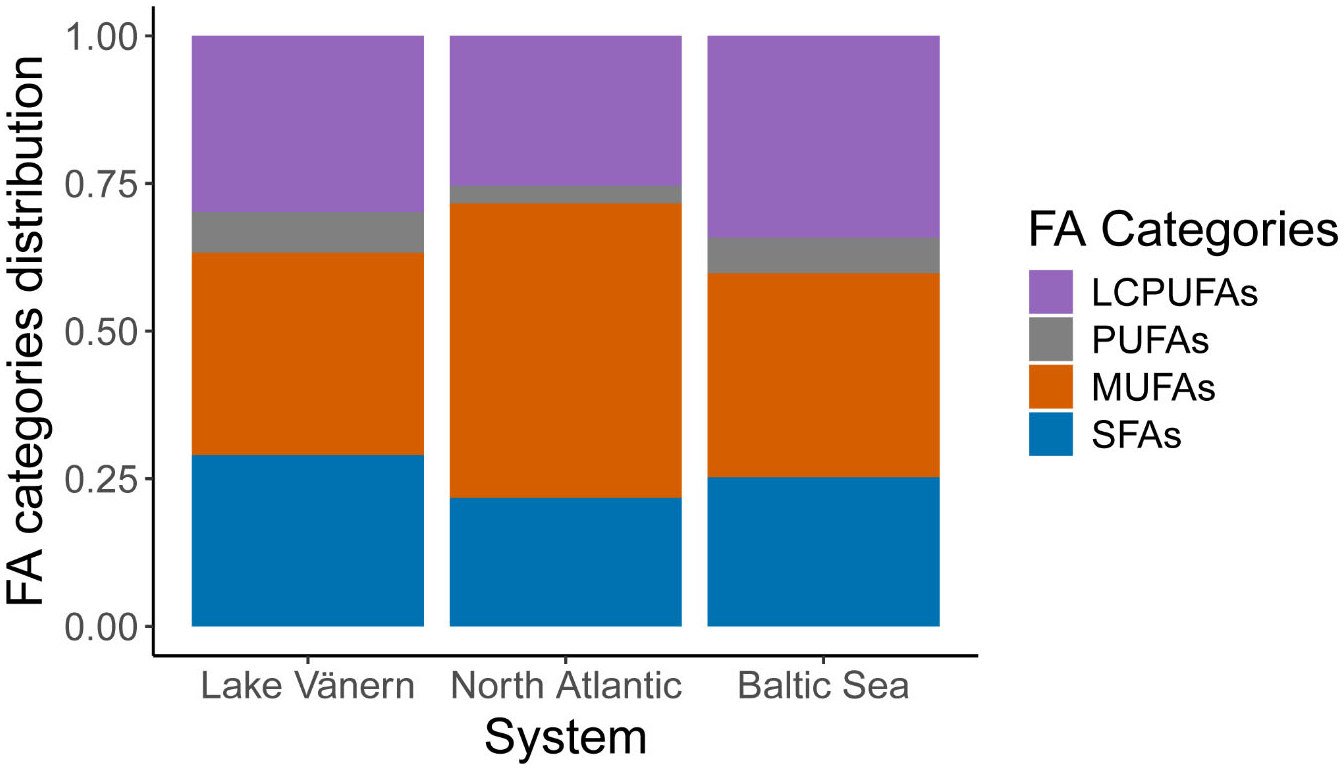
Proportions of the different FA categories in each system for the salmon caught after sea or lake migration.

**FIGURE 3:**
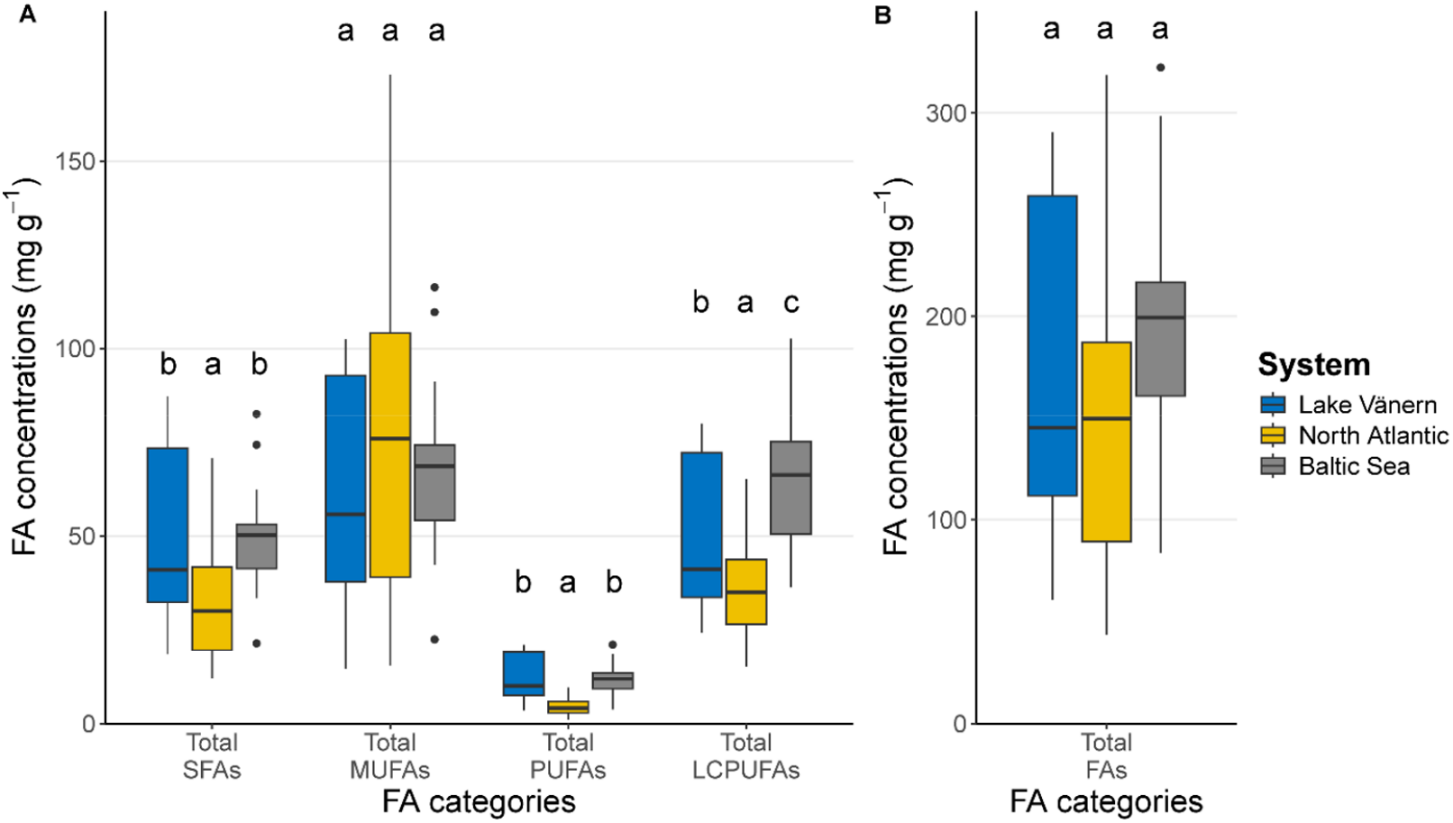
Concentrations of FA structural categories in each system for the samples caught after sea or lake migration. The different letters indicate significant differences across systems within each FA structural category (Tukey post hoc test, *α* = 0.05).

To investigate the differences in FA profiles across the three systems included in the study, i.e. Baltic Sea, North Atlantic and Lake Vänern, we only used samples from the after sea or lake migration life stage (i.e., collected at river mouth locations). This was the only life stage that was collected in all three systems and therefore comparable. The PERMANOVA revealed a significant effect of system on the FA profile composition in muscle samples (PERMANOVA, *F*_2,60_ = 168.82, *p* < 0.001) (Figure 1A). Rivers within the same system—i.e. Drammen and Driva rivers in the North Atlantic Ocean system, and Lule, Torne, and Ume rivers in the Baltic Sea system—showed overlapping clusters (Figure 1B). The same pattern was observed when assessing samples from the actively spawning life stage (Appendix S2). The FA profiles of North Atlantic salmon were characterized mostly by long‐chain MUFAs, representing a larger proportion of the FA composition when comparing the different FA structural categories (Figure 2). However, there was no difference in the concentration of total MUFAs among the three systems (Figure 3). The Lake Vänern FA profiles, and to some extent Baltic Sea FA profiles as well, was characterized by a higher proportion of omega‐6 FAs (Figure 1) and the total omega‐6 FAs concentration was the highest in Lake Vänern when comparing the three systems (for the full list of FAs please see Appendix S3). DHA, which belongs to the LCPUFA structural category, was of greater importance in the FA profiles of Baltic Sea salmon (Figure 1, Appendix S3) compared to the other systems. Hence, total LCPUFA proportion and concentration was greater in the Baltic Sea populations compared to North Atlantic and Lake Vänern ones (Figures 2 and 3, Appendix S3).

**FIGURE 4:**
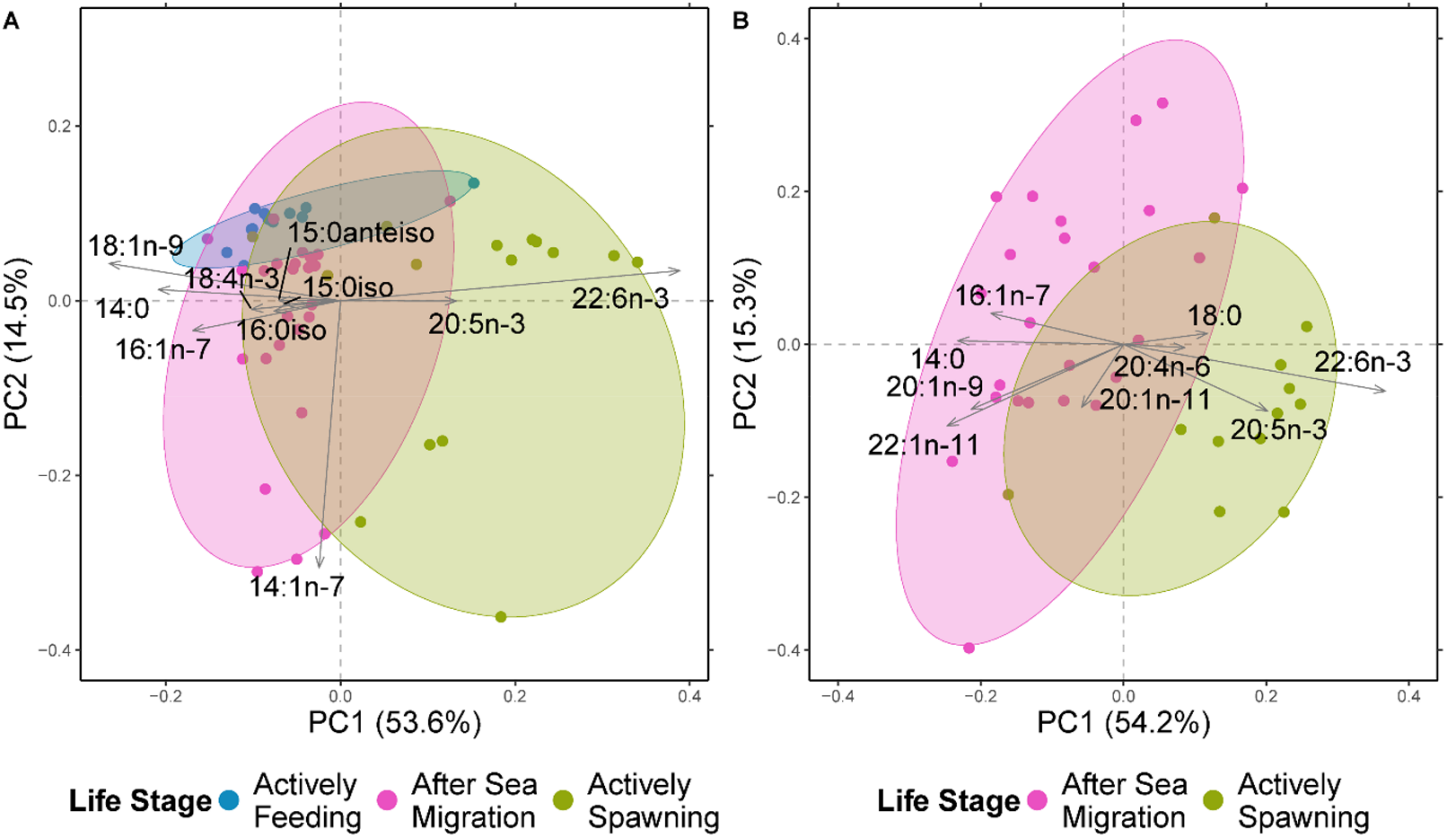
Principal component analysis (PCA) for the (A) Baltic Sea system (*n* = 52) and (B) North Atlantic system (*n* = 36) with principal components 1 and 2 and their respective percentage of the explained variance and they are indicated by the light gray arrows. The three life stages are shown in different colors. The reported FAs explain at least 70% of the variation in the distribution of the samples and they are indicated by the light gray arrows.

When assessing the FA profile during different adult life stages of salmon (i.e., feeding, after sea/lake migration, spawning), only Baltic Sea and North Atlantic systems were considered because spawning fish were not sampled for Lake Vänern (Figure 4). There was a significant effect of life stage on the FA profile composition in muscle samples from both Baltic Sea (PERMANOVA, *F*_2,49_ = 19.24, *p* < 0.001) and North Atlantic (PERMANOVA, *F*_1,34_ = 20.61, *p* < 0.001) salmon (Figure 4). The actively feeding life stage was available only for the Baltic Sea system and its FA profile overlapped with the after sea migration life stage (Figure 4A). Hence, life stages that are closer in time were characterized by similar muscle FA profiles in salmon. Although not complete, there was a separation of the actively spawning life stage to the other life stages with its FA profile being characterized by longer and more unsaturated FAs, such as 20:5n‐3 (EPA) and 22:6n‐3 (DHA) (Figure 4). The same pattern was observed for both the Baltic Sea (Figure 4A) and North Atlantic populations (Figure 4B).

### 3.2 Relationship between Fatty Acids and Thiamine Concentrations

**FIGURE 5:**
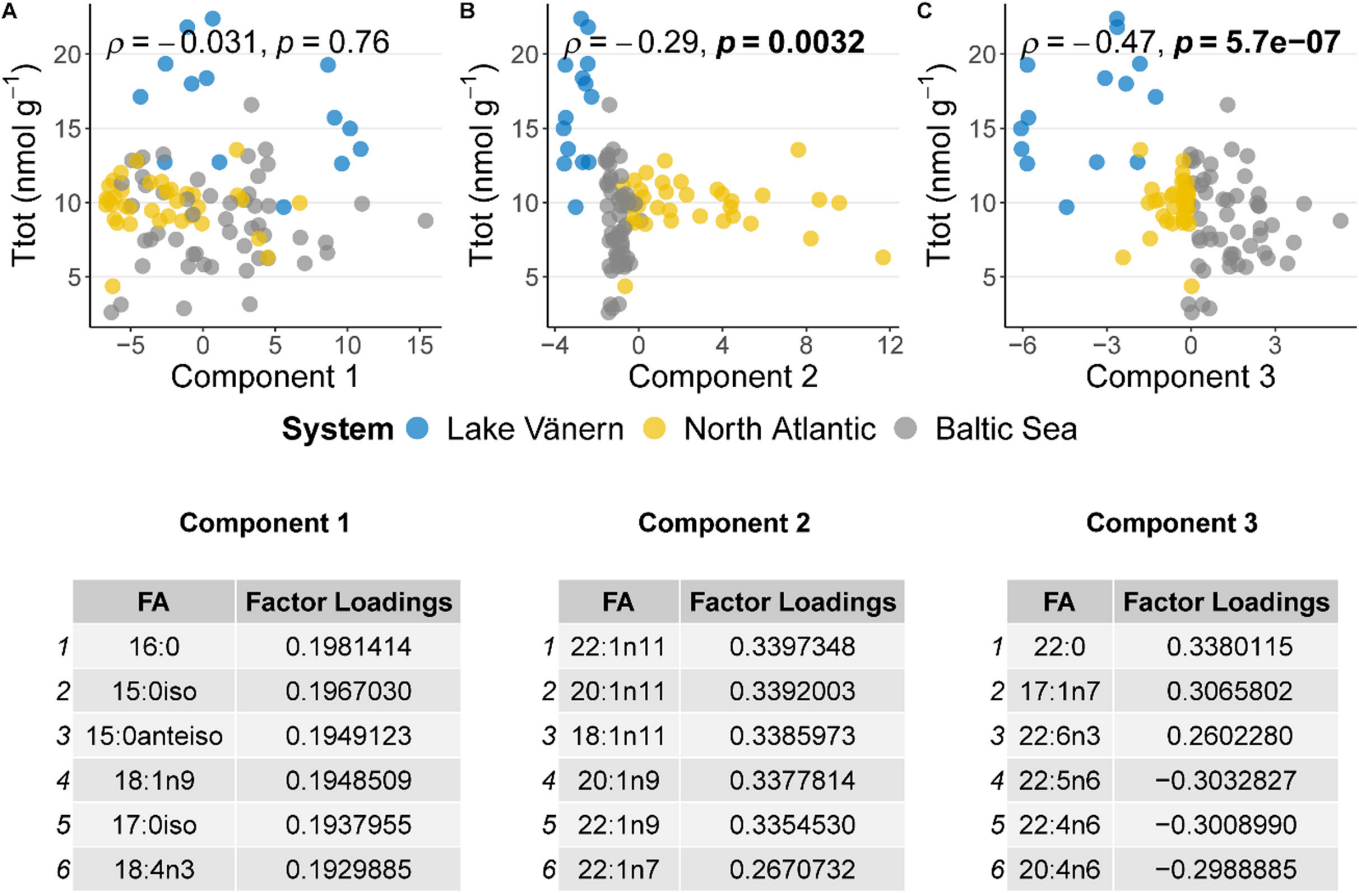
Spearman's correlation between total thiamine concentration in the muscle (Ttot, nmol g^−1^) and (A) Component 1, (B) Component 2, and (C) Component 3 from the PCA (scores) which includes all the samples. Correlation coefficients rho (*ρ*) and *p* values (*p*) are reported. The six FA that had the highest relative principal component factor loadings for each component are displayed in the tables (i.e., these can be interpreted as how much each original variable contributes to the component).

**FIGURE 6:**
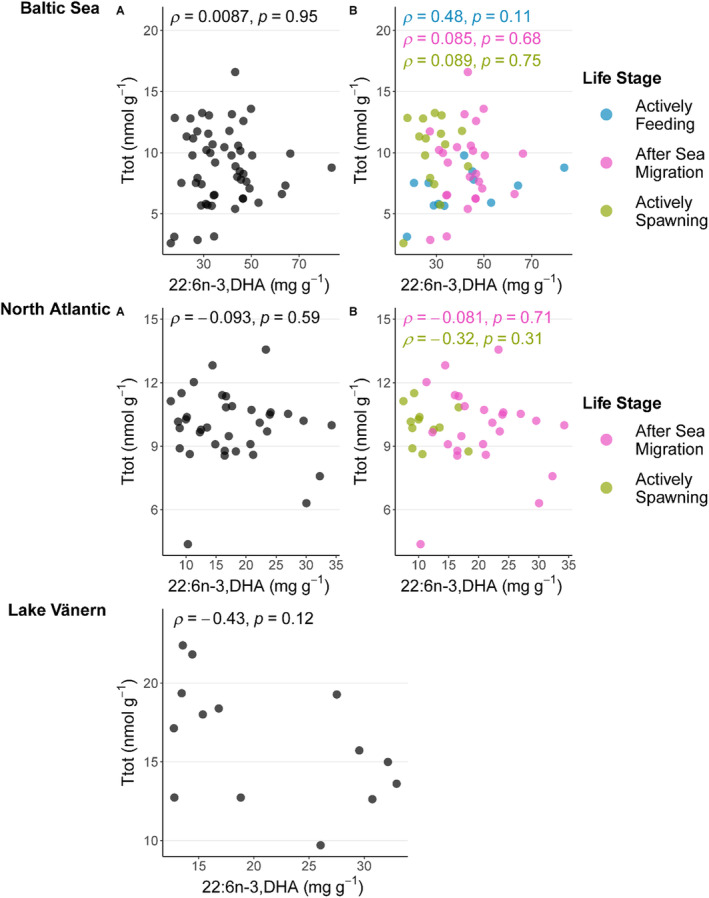
Spearman's correlation between total thiamine concentration in the muscle (Ttot, nmol g^−1^) and DHA (22:6n3, mg g^−1^) in Baltic Sea (*n* = 52) at the top, North Atlantic (*n* = 36) in the middle, and Lake Vänern (*n* = 14) at the bottom. The A panels report the samples all together for each system, the B panels display the samples by life stages indicated by different colors within each system.

The thiamine status of salmon from the three systems, Lake Vänern, North Atlantic and Baltic Sea is generally characterized by high (17.20 ± 3.41 nmol g^−1^), intermediate (10.60 ± 2.31 nmol g^−1^) and low (8.62 ± 4.25 nmol g^−1^) thiamine concentrations in muscle, respectively (Todisco et al. 2024). In order to investigate potential relationships between total thiamine concentration (Ttot) and salmon FA profiles in muscle, all the samples were included in a single PCA (Appendix S4), and the first three principal components were examined (Figure 5). Component 1 explained 59.9% of the variance but it did not correlate with Ttot (Figure 5A). Component 2 explained 20.1% of the variance and there was a significant, albeit weak, negative correlation with Ttot (Figure 5B). Component 3 explained 10.9% of the variance and had a significant negative correlation with Ttot (Figure 5C). The FAs that were positively correlated with Component 3 (highest factor loading values) were 22:0, 17:1n‐7 and 22:6n‐3 (Figure 5C). These FAs were also among the most influential FA separating the relatively thiamine poor Baltic Sea populations from Lake Vänern and North Atlantic populations (Figures 1A and 5C, Appendix S2 panel A). The FAs that were negatively correlated with Component 3 (highest negative factor loading values), were 22:5n‐6, 22:4n‐6, and 20:4n‐6 and they were related to high Ttot and the Lake Vänern system (Figures 1A and 5C). Hence, the correlation between Ttot and Component 3 was mainly driven by differences among systems in terms of thiamine concentration and this suggests that certain FAs might be related to high or low thiamine status (Figure 5C). To further investigate the potential relationships of the above‐mentioned FAs with low thiamine status, the FAs and systems were examined separately, and no correlations were found (Appendix S5). DHA (22:6n‐3) was studied in more detail (Figure 6). No correlation was found in any of the three systems, neither when all the samples within the system were included together (Figure 6, A panels) nor when the samples were separated by life stages (Figure 6, B panels). There were also no negative correlations between TF and DHA in any of the systems and life stages (Appendix S6). In fact, there were instead significant positive correlations between TF and DHA in both Baltic Sea and North Atlantic systems when all the samples were included together (Appendix S6, A panels).

## 4 Discussion

This study found that salmon feeding in various systems (freshwater, brackish, and marine) with differences in overall thiamine status exhibited distinct FA profiles corresponding to their different diets (Figure 1). However, no correlations were found within these systems between different FAs (including DHA) and thiamine status (Figures 5 and 6). This implies that a diet rich in easily oxidized FAs does not necessarily result in reduced thiamine concentration in salmon tissues and thiamine deficiency, as previously hypothesized. Moreover, the salmon FA profiles varied throughout their life cycle (Figure 4). When comparing low‐thiamine fish from the Baltic Sea and medium‐thiamine fish from North Atlantic Ocean, we observed similar FA profile changes from arrival at the spawning river to the reproductive phase, suggesting that these changes are unrelated to thiamine deficiency. Thiamine concentrations have been shown to decline in a similar manner in both of these systems during pre‐spawning fasting suggesting that these changes are likewise unrelated to thiamine deficiency (Todisco et al. 2024).

### 4.1 Systems with Different Overall Thiamine Statuses Have Different FA Profiles

The three systems included in the study, i.e., Lake Vänern (freshwater), Baltic Sea (brackish), and North Atlantic Ocean (marine), had significantly different FA profiles. To some extent, the FA profiles of fish tissues reflects their diet history (Watanabe 1982; Steffens 1997; Xu et al. 2020; Glencross et al. 2024). Lake Vänern FA profiles were most characterized by omega‐6 FAs such as 20:4n‐6, 18:2n‐6, 22:4n‐6, and 22:5n‐6 which indicate a freshwater diet (Ackman 1967; Steffens 1997). Landlocked salmon in Lake Vänern mainly feed on European smelt (*Osmerus eperlanus*) and vendace (*Coregonus albula*) (Nyquist 2022). These two fish species are known to have a relatively high level of 20:4n‐6 (Muje et al. 1989; Linko, Rajasilta, and Hiltunen 1992; Dgebuadze et al. 2023) which is consistent with the FA profiles found in this study. To a lesser degree, the Baltic Sea system FA profile was also characterized by some omega‐6 FAs, especially 18:2n‐6 and 20:2n‐6 in line with the brackish nature of this basin and the related origin of salmon prey species. In other words, the proportion of omega‐6 FAs gives information on which type of ecosystem—freshwater/brackish or marine—the fish was living in while feeding.

One of the FA with the highest concentration in the Baltic Sea salmon FA profiles was DHA (22:6n‐3). This is a common omega‐3 FA in fish in general because it is highly conserved through the food webs up to higher trophic levels due to its incorporation into cell membrane phospholipids (Scott et al. 2002; Dalsgaard et al. 2003). The composition of phytoplanktonic community significantly influences the availability of certain FAs for higher trophic levels since FAs synthesis varies among different phytoplankton taxa (Taipale et al. 2013). For example, freshwater systems are more abundant in green algae and cyanobacteria which are important synthesizers of a shorter omega‐3 FA, the 18:3n‐3 (ALA, α‐linoleic acid, Taipale et al. (2013)). To some extent, freshwater fish and salmonids can use ALA to endogenously synthesize DHA through a series of desaturation and elongation reactions where EPA serves as an intermediate step (Watanabe 1982; Turchini et al. 2022). Another factor that can possibly influence FA productivity is eutrophication (Calderini et al. 2023). It has been demonstrated that the phytoplanktonic production of EPA and DHA, and therefore their availability for higher trophic levels, is higher in meso‐eutrophic than oligotrophic conditions (Calderini et al. 2023). In all, this could potentially explain why DHA was a predominant FA in salmon FA profiles from the Baltic Sea (brackish, eutrophic) compared to Lake Vänern (freshwater but oligotrophic). Furthermore, some of the FAs characterizing the actively feeding and after sea migration life stages in the Baltic Sea included 18:1n‐9, which is one of the FA indicative of a diet that includes sprat (*Sprattus sprattus*) (Keinänen et al. 2017), as well as 14:0, which is indicative for feeding on herring (*Clupea harengus*) in the Southern Baltic Sea (Lind, Huovila, and Käkelä 2018). In contrast, a major proportion of the North Atlantic salmon FA profile was represented by long‐chain MUFAs, such as 22:1n‐11, 20:1n‐9, 20:1n‐11, and 22:1n‐9. These MUFAs can be an indication of direct consumption of lipid‐rich zooplankton, such as *Mysis* or *Calanus* genera (Dalsgaard et al. 2003; Brett, Müller‐Navarra, and Persson 2009), as well as feeding on zooplanktivorous fish (Stowasser, Pond, and Collins 2009). In all, this strongly suggests that salmon from the three different systems have distinct diet histories and related FA profiles. Salmon from the systems included in this study are also known to also have different thiamine status, that is low, intermediate and high in the Baltic Sea, North Atlantic and Lake Vänern, respectively (Todisco et al. 2024). The FA profiles described in this study suggest that this corresponds to diets with high levels of piscivory, mixed diets and high levels of piscivory for the Baltic Sea, North Atlantic, and Lake Vänern salmon, respectively. Hence, a highly piscivorous diet is, on its own, not a good predictor of high or low thiamine status.

### 4.2 A Diet Rich in Easily Oxidized FAs Does Not Necessarily Result in Reduced Thiamine Concentrations in Salmon Tissues

Gut content analyses suggest that salmon in the Baltic Sea feed mostly on sprat and herring (Karlsson et al. 1999; Hansson et al. 2001) and thiamine deficiency has been suggested to be associated with a lipid‐rich diet with a high proportion of DHA (Keinänen et al. 2012, 2017, 2018, 2022; Vuorinen et al. 2024). Especially small sprat have been shown to have this FA profile, where the thiamine concentration in sprat varies approximately between 5 and 6 nmol g^−1^ along a DHA range of approximately 14–15 mg g^−1^ (Keinänen et al. 2017). However, there are also examples of thiamine‐deficient salmon that have herring‐dominated diet (as inferred by FA signatures; Keinänen et al. (2018); Keinänen et al. (2022)). Keinänen et al. (2022) argues that the negative effect of a lipid‐rich diet on thiamine status is not dependent on the prey species per se but rather their FA profiles, suggesting that consumption of sprat, herring or other lipid‐rich prey items can cause thiamine deficiency. The proposed mechanisms for lipid‐rich diets causing thiamine deficiency are: (1) thiamine requirements increase when feeding on high‐energy food items and; (2) thiamine is used as an antioxidant defense against oxidative stress when high levels of DHA are metabolized (Keinänen et al. 2012, 2017, 2022). DHA is a highly unsaturated omega‐3 FA which is known to be particularly vulnerable to lipid peroxidation (Kjær et al. 2008). Keinänen et al. (2022) indeed found a negative relationship between free thiamine (TF) in salmon muscle and the total lipid proportion and DHA concentration. This was coupled with a positive correlation between DHA and hepatic malondialdehyde, a biomarker which is indicative of oxidative stress in salmon and a product of lipid peroxidation (Keinänen et al. 2022). We found that the Baltic Sea fish had the highest concentrations of DHA of the three systems. Concentrations were in the higher range of concentrations previously observed in thiamine depleted fish (Keinänen et al. 2022), but we did not find any negative correlations between DHA and Ttot nor with TF, which is in contrast to previous studies (Keinänen et al. 2022). This indicates that additional factors, besides a high DHA concentration, are the cause of thiamine deficiency in salmon.

The potential relationship between thiamine status and lipids and especially DHA has also been assessed in a range of salmonid species in the North American Great Lakes (Futia et al. 2019). Similar to this study, Futia et al. (2019) found little evidence of correlations between thiamine and lipid or DHA content. There were negative associations in brown trout (*Salmo trutta*) which is typically not affected by thiamine deficiency, but no such correlations among other salmonids that had lower thiamine concentrations (Futia et al. 2019). The same salmonid species in Lake Superior also have similar lipid contents as thiamine‐deficient fish in other Great Lakes without developing thiamine deficiency (Miller and Schram 2000; Neff, Bhavsar, and Chin 2012; Futia et al. 2019).

Even during spawning, the adult life stage where thiamine levels have been observed to be lower than other adult life stages (Todisco et al. 2024), and during which the FA profile is dominated by DHA, there was no correlation between DHA and thiamine concentrations. Other studies have found an increase in biochemical markers (hepatic malondialdehyde) indicating oxidative stress in salmon preparing for spawning (Vuorinen et al. 2020) and this has been interpreted as a sign that thiamine has been used as an antioxidant. Here, both Baltic Sea salmon, known to exhibit thiamine deficiency, and North Atlantic salmon, which are not known to exhibit thiamine deficiency, had similar changes in their FA profiles with both being richer in omega‐3 FAs toward spawning. Hence, as we see this accumulation of easily oxidized FA in both Baltic and North Atlantic salmon, it may be a natural consequence of preparation for spawning and not a process specifically related to thiamine deficiency.

### 4.3 Thiamine's Potential Role as Primary Antioxidative Defense in Fish Cells

While it is agreed that thiamine possesses antioxidant properties (Lukienko et al. 2000), its role as a primary defense against oxidative stress remains uncertain. Lukienko et al. (2000) examined these properties in the presence of oleic acid (18:1n‐9) peroxidation in rat liver cells. However, the role of omega‐3 FAs, which are often hypothesized to be implicated in thiamine deficiency in salmonids (Keinänen et al. 2022), remains unexplored as well as thiamine antioxidant properties in fish cells in general. Moreover, numerous other potent antioxidant systems exist in living cells, which can be induced by oxidative stress e.g., superoxide dismutase, glutathione peroxidase, glutathione‐S‐transferase, α‐tocopherol, catalase, and vitamin C, all of which are present in fish cells (Sargent, Tocher, and Bell 2003; Van der Oost, Beyer, and Vermeulen 2003; Martínez‐Álvarez, Morales, and Sanz 2005; Olsvik et al. 2005). This suggests that for thiamine to be used as an antioxidant, a cell or organism would need to be depleted of other more potent antioxidant systems, in case these systems are used as preferential antioxidants before thiamine. This questions the hypothesis that thiamine deficiency is caused by thiamine depletion due to oxidative stress. Furthermore, the antioxidant efficacy of thiamine does not appear to be effective at high concentrations. For instance, in rat liver cells exposed to lead‐induced oxidative stress, the most effective was an intermediate concentration of vitamin C and the lowest concentration of thiamine (Wang et al. 2007). This suggests that the efficiency of thiamine as an antioxidant is concentration‐dependent, and the most beneficial effect may not be observed at its highest concentration in the presence of more potent antioxidants such as vitamin C. Hence, although some studies find negative correlations between thiamine and omega‐3 FAs (Keinänen et al. 2022), more research is needed to demonstrate that the oxidative stress imposed by metabolism of omega‐3 FAs indeed happens in fish cells and at rates that could lead to thiamine deficiency.

### 4.4 Other Factors That Might Affect the Thiamine Status of Salmon

Hence, there is mixed evidence for the hypothesis that a high fat diet leads to thiamine deficiency and this study suggests that other mechanisms might be of greater importance. It may be possible that systems with a low thiamine status, like the Baltic Sea, have fewer thiamine‐rich prey species. For example, zooplankton are in the order of 2–10 times richer per carbon unit in thiamine compared to small fish (Hylander et al. in press; Fridolfsson et al. (2019); Fridolfsson et al. (2020)). It has also been demonstrated that a more diverse diet promotes higher thiamine concentrations in predators in the Great Lakes of North America (Futia et al. 2019). This might explain why the North Atlantic system, where salmon have a more mixed diet composed by both fish and crustaceans (Hansen and Olafsen 1999; Jacobsen and Hansen 2000; Haugland et al. 2006), had an intermediate thiamine status and, so far, no record of thiamine deficiency (Todisco et al. 2024). However, salmon thiamine concentration was, on average, the highest in Lake Vänern where the FA profile indicated high levels of piscivory demonstrating that a high thiamine concentration can be maintained without feeding on lower trophic levels such as large zooplankton and macroinvertebrates.

Furthermore, in other systems such as the Great Lakes in North America, thiamine deficiency has been associated with the presence of thiaminase I, a thiamine‐degrading enzyme, derived from salmonid prey items (Fisher et al. 1995, 1996; Fitzsimons et al. 2005; Honeyfield et al. 2005). In the Baltic Sea, thiaminase activity has been observed in the salmon main prey items, both in sprat and herring (Wistbacka, Heinonen, and Bylund 2002; Wistbacka and Bylund 2008). However, it is not known if prey items contributing to thiaminase I activity are more common in the Baltic Sea compared to Lake Vänern or the North Atlantic systems. Interestingly, recent studies show that prey items that have potential thiaminase I activity are also rich in omega 3‐FAs (Rowland et al. 2023). Hence, future research should investigate whether it is the fat content, the thiaminase I activity, or some other factor that causes thiamine deficiency in the top‐consumer fish.

## 5 Conclusions

This study has provided a comprehensive examination of the relationship between thiamine status and diet, specifically through exploring the FA profiles of salmon from three different systems. The findings reveal that salmon, with varying thiamine levels, feeding in different systems, displayed unique FA profiles, reflecting their diverse diets. More importantly, no correlation was found between different FAs, including DHA, and thiamine status within these systems. This suggests that a diet rich in easily oxidized FAs does not necessarily lead to lower thiamine concentration in salmon tissues, in contrast with previous hypotheses. Furthermore, salmon FA profiles were found to vary throughout their adult life cycle. When comparing low‐thiamine brackish populations with medium‐thiamine marine ones, similar FA profile changes were observed from their arrival at the spawning river to the reproductive phase. This indicates that these changes are not linked to thiamine deficiency but might rather be the natural consequence of starvation and preparation for spawning. Finally, this study leads to the conclusion that thiamine's function as an antioxidant should be reassessed in mechanistic experiments quantifying the speed of potential thiamine depletion in fish cells when challenged with oxidative stress from DHA. This would test whether this mechanism is sufficiently strong to cause thiamine deficiency or if other additional factors, as suggested by this study, need to occur for thiamine deficiency to develop.

## Author Contributions

**Vittoria Todisco:** conceptualization (lead), data curation (lead), formal analysis (lead), investigation (lead), methodology (lead), validation (lead), visualization (lead), writing – original draft (lead), writing – review and editing (lead). **Marc M. Hauber:** data curation (supporting), formal analysis (supporting), investigation (supporting), methodology (supporting), validation (supporting), writing – review and editing (equal). **Michael T. Brett:** conceptualization (supporting), data curation (supporting), formal analysis (supporting), methodology (lead), resources (supporting), supervision (supporting), validation (equal), writing – review and editing (equal). **Charlotte Axén:** funding acquisition (supporting), investigation (lead), methodology (supporting), project administration (equal), resources (supporting), supervision (supporting), validation (supporting), writing – review and editing (equal). **Kjetil Hindar:** conceptualization (supporting), investigation (lead), project administration (supporting), resources (supporting), supervision (supporting), validation (supporting), writing – review and editing (equal). **Petter Tibblin:** conceptualization (supporting), formal analysis (supporting), funding acquisition (supporting), investigation (supporting), methodology (equal), project administration (supporting), resources (supporting), supervision (supporting), validation (supporting), writing – review and editing (equal). **Samuel Hylander:** conceptualization (lead), data curation (equal), formal analysis (equal), funding acquisition (lead), investigation (supporting), methodology (equal), project administration (lead), resources (lead), supervision (lead), validation (lead), visualization (equal), writing – original draft (equal), writing – review and editing (equal).

## Conflicts of Interest

The authors declare no conflicts of interest.

## Supporting information


Data S1.

## Data Availability

All the raw data is available in the Supporting Information.
