# Supplementary material for: The Implications of Atlantic Salmon (Salmo salar L.) Fatty Acid Profiles for Their Thiamine Status
Source: Ecol Evol. 2024 Oct 25;14(10):e70478. doi: 10.1002/ece3.70478 (PMC11511624; doi:10.1002/ece3.70478)
Supplement: Supplementary file 1 — Data S1. [file ECE3-14-e70478-s001.zip › All appendices 1 through 6.docx]

# Appendices

| **System** | **Area** | **Site** | **Number of individuals** | **Weight**  **(mean ± SD)** | **Total Length**  **(mean ± SD)** | **CF**  **(mean ± SD)** |
| --- | --- | --- | --- | --- | --- | --- |
| Baltic Populations | Southern Baltic Sea |  | 12 | 4.17 ± 1.64 | 75.54 ± 10.17 | 0.90 ± 0.11 |
|  | Torneälven | River mouth | 11 | 8.71 ± 2.86 | 93.23 ± 8.91 | 1.04 ± 0.08 |
|  | Luleälven | River mouth | 11 | 7.95 ± 1.81 | 92.04 ± 7.66 | 1.00 ± 0.07 |
|  |  | Upstream | 15 | 7.12 ± 1.45 | 92.47 ± 6.12 | 0.89 ± 0.10 |
|  | Umeälven | River mouth | 3 | 6.24 ± 0.72 | 87.67 ± 3.51 | 0.92 ± 0.04 |
| North Atlantic Populations | Drammen | River mouth | 11 | 5.20 ± 0.87 | 80.59 ± 4.16 | 0.98 ± 0.05 |
|  |  | Upstream | 8 | 4.54 ± 0.73 | 82.37 ± 3.51 | 0.81 ± 0.09 |
|  | Driva | River mouth | 13 | 4.60 ± 2.00 | 79.23 ± 8.72 | 0.88 ± 0.07 |
|  |  | Upstream | 4 | 4.16 ± 0.76 | 79.00 ± 5.35 | 0.84 ± 0.03 |
| Lake Vänern Population | Lake Vänern | In Lake at river mouth | 14 | 4.64 ± 1.22 | 72.14 ± 5.59 | 1.21 ± 0.14 |

Appendix S1 Means of weight in kg and total length in cm for every sampling occasion, and CF (kg cm^-3^). Standard deviation (SD) is also reported.


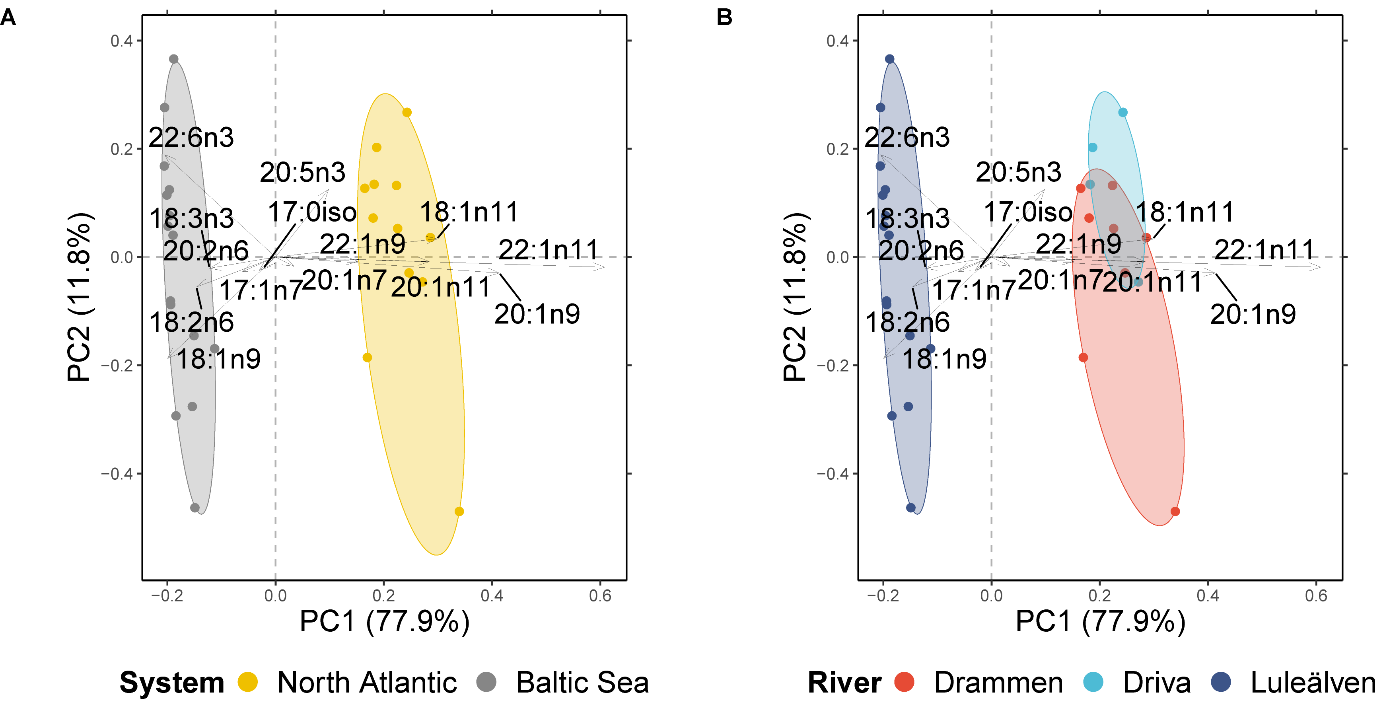


Appendix S2 Principal component analysis (PCA) with principal components 1 and 2 and their respective percentage of the explained variance. The reported FAs explain at least 70% of the sample variation. Only samples from the actively spawning life stage are included in this figure. A) The two systems in different colors are reported for a total n = 27 (PERMANOVA, F_1,25_ = 64.36, p < 0.001); B) A different color indicates each river.

| **FA category** | **FA** | **North Atlantic** | **Baltic** | **Lake Vänern** | ***p*** |
| --- | --- | --- | --- | --- | --- |
| SFAs | 14:0 | 5.21 ± 0.49 | 4.97 ± 0.48 | 4.42 ± 0.68 | 0.611 |
|  | 16:0 | 20.33 ± 2.23 ^a^ | 33.14 ± 2.19 ^b^ | 32.68 ± 2.92 ^b^ | < 0.001 |
|  | 17:0 | 0.58 ± 0.06 | 0.66 ± 0.06 | 0.52 ± 0.08 | 0.340 |
|  | 18:0 | 4.37 ± 0.53 ^a^ | 7.13 ± 0.52 ^b^ | 10.30 ± 0.70 ^c^ | < 0.001 |
| MUFAs | 14:1n-7 | 0.11 ± 0.23 | 0.63 ± 0.22 | 0.53 ± 0.30 | 0.257 |
|  | 16:1n-7 | 7.99 ± 0.96 | 7.90 ± 0.94 | 11.61 ± 1.26 | 0.043 |
|  | 16:1n-9 | 0.26 ± 0.06 ^a^ | 0.76 ± 0.05 ^b^ | 0.86 ± 0.07 ^b^ | < 0.001 |
|  | 18:1n-11 | 2.03 ± 0.16 ^b^ | 0.005 ± 0.16 ^a^ | - | < 0.001 |
|  | 18:1n-9 | 23.22 ± 2.93 ^a^ | 44.86 ± 2.87 ^b^ | 35.60 ± 3.84 ^b^ | < 0.001 |
|  | 18:1n-7 | 5.29 ± 0.60 ^a^ | 6.10 ± 0.59 ^ab^ | 8.00 ± 0.78 ^b^ | 0.029 |
|  | 20:1n-11 | 2.59 ± 0.21 ^b^ | 0.08 ± 0.21 ^a^ | 0.08 ± 0.28 ^a^ | < 0.001 |
|  | 20:1n-9 | 16.09 ± 1.30 ^b^ | 2.63 ± 1.27 ^a^ | 2.18 ± 1.70 ^a^ | < 0.001 |
|  | 22:1n-11 | 15.44 ± 1.21 ^b^ | 0.12 ± 1.18 ^a^ | - | < 0.001 |
|  | 22:1n-9 | 1.96 ± 0.17 ^b^ | 0.38 ± 0.17 ^a^ | 0.25 ± 0.22 ^a^ | < 0.001 |
|  | 24:1n-9 | 1.31 ± 0.13 ^a^ | 2.04 ± 0.13 ^b^ | 1.01 ± 0.18 ^a^ | < 0.001 |
| PUFAs | 18:2n-6 | 1.84 ± 0.35 ^a^ | 5.59 ± 0.34 ^b^ | 4.50 ± 0.46 ^b^ | < 0.001 |
|  | 18:3n-3 | 1.15 ± 0.35 ^a^ | 4.15 ± 0.35 ^b^ | 5.98 ± 0.46 ^c^ | < 0.001 |
|  | 18:4n-3 | 1.59 ± 0.18 ^a^ | 2.15 ± 0.18 | 1.84 ± 0.24 | 0.093 |
| LCPUFAs | 20:2n-6 | 0.52 ± 0.10 ^a^ | 1.42 ± 0.10 ^b^ | 1.22 ± 0.13 ^b^ | < 0.001 |
|  | 20:4n-6 | 0.63 ± 0.21 ^a^ | 1.48 ± 0.20 ^b^ | 5.00 ± 0.27 ^c^ | < 0.001 |
|  | 20:5n-3 | 9.35 ± 0.79 | 11.49 ± 0.78 | 9.57 ± 1.04 | 0.126 |
|  | 22:4n-6 | 0.06 ± 0.08 ^a^ | 0.34 ± 0.09 ^b^ | 1.69 ± 0.10 ^c^ | < 0.001 |
|  | 22:5n-6 | 0.22 ± 0.13 ^a^ | 0.55 ± 0.12 ^a^ | 3.08 ± 0.50 ^b^ | < 0.001 |
|  | 22:5n-3 | 3.99 ± 0.50 ^a^ | 6.82 ± 0.49 ^b^ | 7.17 ± 0.65 ^b^ | < 0.001 |
|  | 22:6n-3 | 20.69 ± 1.68 ^a^ | 42.81 ± 1.64 ^b^ | 21.21 ± 2.19 ^a^ | < 0.001 |
| Total n-3 FAs | | 36.77 ± 3.36 ^a^ | 67.43 ± 3.29 ^b^ | 45.77 ± 4.40 ^a^ | < 0.001 |
| Total n-6 FAs | | 3.27 ± 0.81 ^a^ | 9.39 ± 0.79 ^b^ | 15.49 ± 1.06 ^c^ | < 0.001 |
| Total FAs | | 150.72 ± 13.94 | 193.70 ± 13.66 | 174.47 ± 18.25 | 0.097 |

Appendix S3 Estimated marginal mean concentrations of fatty acids in mg g^-1^ (± SE) in the three systems for the salmon caught after sea or lake migration; n-3, n-6 FA, and total FA are also reported. The p-value in one-way ANOVAs is reported in the “p” column, and the different superscript letters indicate a significant difference across systems (Tukey post hoc test, α = 0.05). The table includes only the fatty acids for which the Area % in the chromatogram was higher than 0.3%.


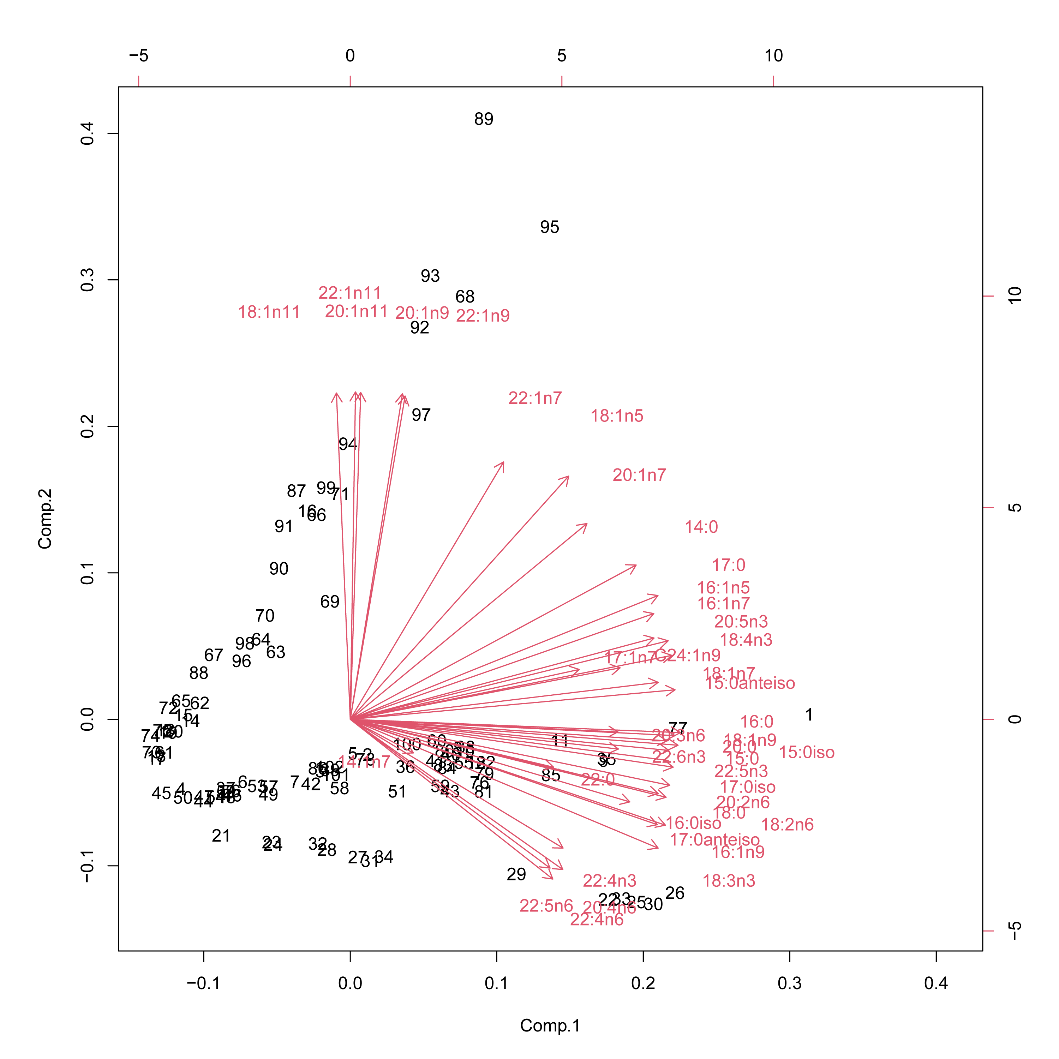


Appendix S4 Principal component analysis (PCA) including all the samples (n=102). The black numbers indicate the samples and the arrows indicate the 39 FAs identified.


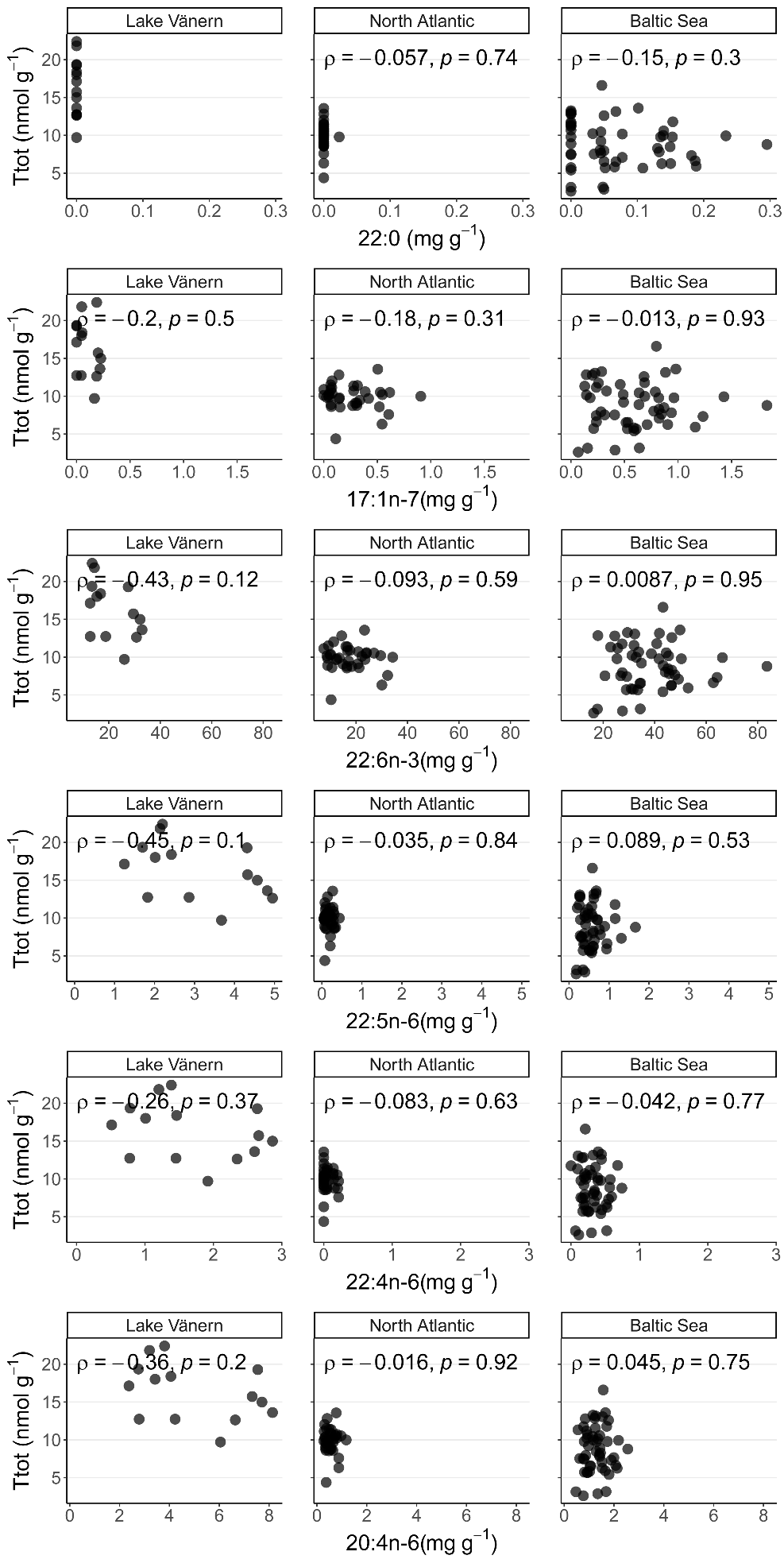


Appendix S5 Spearman’s correlation between total thiamin concentration in the muscle (Ttot, nmol g^-1^) and the positively correlated with Component 3 FAs (22:0, 17:1n7, 22:6n3) and negatively correlated with Component 3 FAs (22:5n6, 22:4n6, 20:4n6) in Lake Vänern (n=14), North Atlantic (n=36), and Baltic Sea (n=52).


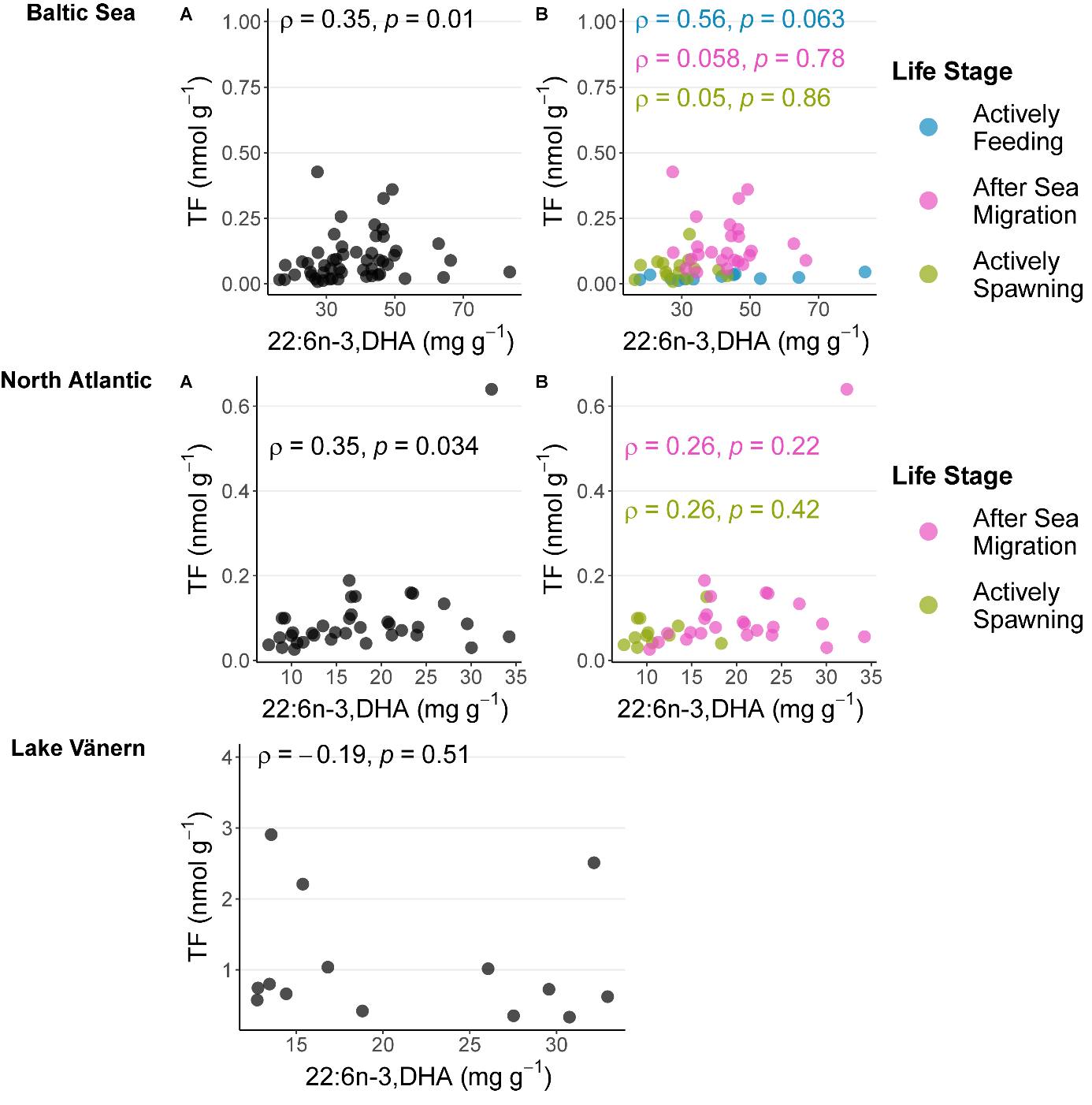


Appendix S6 Spearman’s correlation between free thiamin concentration in the muscle (TF, nmol g^-1^) and DHA (22:6n3, mg g^-1^) in Baltic Sea (n=52) at the top, North Atlantic (n=36) in the middle, and Lake Vänern (n=14) at the bottom. The A panels report the samples all together for each system, the B panels display the samples by life stages indicated by different colors within each system.
