# Supplementary material for: The Implications of Atlantic Salmon (Salmo salar L.) Fatty Acid Profiles for Their Thiamine Status
Source: Ecol Evol. 2024 Oct 25;14(10):e70478. doi: 10.1002/ece3.70478 (PMC11511624; doi:10.1002/ece3.70478)
Supplement: Supplementary file 1 — Data S1. [file ECE3-14-e70478-s001.zip › Supplementary_Todisco et al.docx]

# Supplementary material

# The implications of Atlantic salmon (*Salmo salar*) fatty acid profiles for their thiamine status

## Vittoria Todisco^1^, Marc M. Hauber^1^, Michael T. Brett^2^, Charlotte Axén^3^, Kjetil Hindar^4^, Petter Tibblin^1^, Samuel Hylander^1*^

Affiliations:

^1^ Centre for ecology and evolution in microbial model systems (EEMiS), Linnaeus University, Kalmar, Sweden

^2^ Department of Civil and Environmental Engineering (CEE), University of Washington, Seattle, USA

^3^ Swedish Veterinary Agency (SVA), Uppsala, Sweden

^4^ Norwegian Institute for Nature Research (NINA), Trondheim, Norway

*Corresponding author

Raw data description

The raw data underlying this article is available in a separate file named “Todisco et al raw data”. The file has two sheets where FAconc contains the fatty acid concentrations in the unit mg sample/mg FA. The second sheet is named MetadataThi and contains thiamine concentrations as well as the meta-data including system, life stage, site, fish length and weight. The two sheets can be merged using fishID to get the full dataset underlying the article.
